# A validity-driven approach to the understanding of the personal and societal burden of low back pain: development of a conceptual and measurement model

**DOI:** 10.1186/ar3468

**Published:** 2011-09-20

**Authors:** Rachelle Buchbinder, Roy Batterham, Gerald Elsworth, Clermont E Dionne, Emma Irvin, Richard H Osborne

**Affiliations:** 1 Monash Department of Clinical Epidemiology, Cabrini Hospital, 183 Wattletree Road, Malvern, Victoria 3144, Australia; 2 Department of Epidemiology and Preventive Medicine, School of Public Health and Preventive Medicine, Monash University, The Alfred Centre, 99 Commercial Rd, Melbourne, Victoria 3004, Australia; 3 Public Health Innovation, Faculty of Health, Deakin University, 221 Burwood Highway, Burwood, Victoria 3125 Australia; 4 Population Health Research Unit (URESP), Research Centre of the Laval University Affiliated Hospital, Hôpital du St-Sacrement, 1050 chemin Ste-Foy, Québec, G1S 4L8, Canada; 5 Department of Rehabilitation, Faculty of Medicine, Laval University, Hôpital du St-Sacrement, 1050 chemin Ste-Foy, Québec, G1S 4L8, Canada; 6 Institute for Work & Health, 81 University Avenue, Suite 800, Toronto, Ontario, M5G 2E9, Canada

## Abstract

**Introduction:**

While the importance and magnitude of the burden of low back pain upon the individual is well recognized, a systematic understanding of the impact of the condition on individuals is currently hampered by the lack of an organized understanding of what aspects of a person's life are affected and the lack of comprehensive measures for these effects. The aim of the present study was to develop a conceptual and measurement model of the overall burden of low back pain from the individual's perspective using a validity-driven approach.

**Methods:**

To define the breadth of low back pain burden we conducted three concept-mapping workshops to generate an item pool. Two face-to-face workshops (Australia) were conducted with people with low back pain and clinicians and policy-makers, respectively. A third workshop (USA) was held with international multidisciplinary experts. Multidimensional scaling, cluster analysis, participant input and thematic analyses organized participants' ideas into clusters of ideas that then informed the conceptual model.

**Results:**

One hundred and ninety-nine statements were generated. Considerable overlap was observed between groups, and four major clusters were observed - Psychosocial, Physical, Treatment and Employment - each with between two and six subclusters. Content analysis revealed that elements of the Psychosocial cluster were sufficiently distinct to be split into Psychological and Social, and a further cluster of elements termed Positive Effects also emerged. Finally, a hypothesized structure was proposed with six domains and 16 subdomains. New domains not previously considered in the back pain field emerged for psychometric verification: loss of independence, worry about the future, and negative or discriminatory actions by others.

**Conclusions:**

Using a grounded approach, an explicit *a priori *and testable model of the overall burden of low back pain has been proposed that captures the full breadth of the burden experienced by patients and observed by experts.

## Introduction

Low back pain affects 80 to 85% of people at some stage in their life [1,2] and is a major source of morbidity throughout the world [3]. This condition is one of the most common causes of disability, lost work-days and visits to primary care practitioners in high-income countries [4-8]. Not only does low back pain have physical, psychological, social and economic consequences on the individual, its impact upon families, communities, industries and governments is enormous [4,9,10]. Recent epidemiological studies indicate that severe low back pain increases into old age [9] and may be increasing in prevalence in adolescence [11,12], demonstrating a growing public health concern [13].

While the importance and magnitude of the burden of low back pain upon the individual is well recognized, a systematic understanding of the impact of the condition on individuals is currently hampered by the lack of an organized understanding of what aspects of a person's life are affected, and also by the lack of comprehensive measures of these effects. The burden of a disease is commonly defined in terms of mortality, morbidity (incidence and prevalence), cost and, more recently, disability and quality of life. While these are recognized as components of disease burden, none alone are sufficient for quantifying the overall burden of low back pain from the perspective of the individuals affected.

To date the measurement of the burden of low back pain has been based on indicators such as those mentioned above rather than on empirical reflections of the way in which back pain affects the lives of individuals with the condition and those associated with them. In part this relates to a general problem in measurement development, where measures are often based on theory or historically convenient indicators and tools. Measures developed using this process rarely provide a complete view of an issue and they are usually incomplete in unknown ways. The psychometric literature refers to the failure to cover all aspects of an issue as 'construct under-representation' [14], and highlight this as a serious threat to the validity of any measurement tool [14,15].

The greater danger is that measures based upon incomplete coverage of a problem may then become widely used, which in turn affects the care provided and the outcomes that are valued (and funded). In relation to back pain, there is a mismatch between traditional approaches to measurement of impact, which have little focus on social issues, and evidence showing that social issues and complex interactions between social, psychological, physical and functional issues are the norm [16,17].

The present paper has two equal and interacting aims. First, the article aims to develop a conceptual framework that can be generalized cross-culturally, to estimate the various impacts and overall burden of low back pain from the perspective of individuals with this condition and to explore the pathways by which the individual burden of low back pain becomes a burden for society. This conceptual model will then guide the development of the new measure.

The second aim of the paper is to demonstrate, using the example of low back pain, a process for concept definition and instrument development that is consciously and deliberately directed by modern approaches to validity, from the initial stages of conceptualization through all stages of application of the resultant tool.

In trying to capture these interacting aims, we have adopted the term validity-driven to describe a process that includes: grounded approaches to a concept definition that includes consultation with a broad range of stakeholders and deliberately eschews prevailing theories until later in the development process; stakeholder participation in the organization of ideas into groups that form the basis for hypothesizing scales to be included in the measurement tool; the development of *a priori *hypotheses about the way in which items co-vary and can be used to form measurement scales; recognition that construct validation is an ongoing process, and that an instrument is never validated but that each interpretation of the scores needs to be validated; and the specification of a program of research to support the valid application of the tool in relation to an increasing range of interpretations (uses).

In keeping with this process, the end point of the present paper is the detailing of the hypothesized measurement model of the overall burden of back pain from the perspective of individuals with this condition and the description of a proposed program of validation research. The approaches described in this paper have evolved in the instrument development and application work of members of the research team over more than a decade [18-24]. However, this is the first time that the whole process was formalized in advance, as a comprehensive approach to instrument development.

## Materials and methods

### Study design and participants

A grounded approach to conceptualization and the identification of draft items maximizes the likelihood that the resultant tool will fully cover the construct; in this case, the burden of low back pain. Our process for grounded conceptualization included three concept mapping groups that utilized processes modified from the methods developed by Trochim [25]. Concept mapping is a formal group process tool for identifying and organizing ideas on a topic of interest. The steps include development of a seeding statement, generation of statements (brainstorming), sorting of the statements, generation of a concept map and revision of the concept map.

The Cabrini Human Research Ethics Committee approved the study (No. 13-02-03-09) and all patients who participated in the study provided written informed consent.

### Naming groups of items that are (or are hypothesized to be) related

There are many options for naming groups of items, including clusters, domains, factors, scales and dimensions. We chose not to use the term 'dimensions' because it has a specific meaning when using multidimensional scaling (MDS), which relates to the number of spatial dimensions in which the MDS software seeks to fit the distances between items. We also chose not to use the term 'factors' because it relates to a specific type of statistical technique - factor analysis.

We use the term 'clusters' when we refer to the outcomes of concept mapping and the term 'domains' when we refer to a refined, hypothesized structure for a proposed instrument. These domains are referred to technically as latent variables during psychometric analysis using structural equation modeling. We use the term 'scales' after the psychometric properties of the instrument have been established.

We consider that the matching between clusters, domains (latent variables) and scales is one of the critical elements in demonstrating construct validity of the final tool. We also use the term 'statements' to refer to the ideas generated by participants in the concept mapping groups, and use the term 'items' when we have begun to redraft these statements into a form that is suitable for a questionnaire.

### Concept mapping workshops with patients and professionals

We conducted two face-to-face concept mapping workshops in Melbourne, Australia. We sought patients from typical clinical and community settings, with the intention of capturing a broad range of experiences. One workshop included patients with low back pain of varying duration and severity recruited from a community-based rheumatology private practice as well as individuals who had identified themselves as having back pain from a research database of people with chronic conditions who have participated in chronic disease self-management education programs across Australia, held at the Centre for Rheumatic Diseases, University of Melbourne (*n *= 8).

The other workshop included a diverse range of clinicians and health policy-makers from government, WorkSafe (a government-operated workers' compensation insurance scheme in Victoria, Australia) and private health insurers, identified through professional networks and snowball recruitment (*n *= 10). We separated the patient and professional groups in order to facilitate frank discussion, and broad and rapid brainstorming.

To maximize the richness and depth of the data obtained, we used a nominal group process that is a method for obtaining the most comprehensive possible range of ideas from individuals on a topic of interest [26]. Usual practice in qualitative data collection is to sample to saturation, which is the point at which no new ideas are emerging. The concept mapping process goes to great lengths to be as exhaustive as possible within each group, and therefore saturation is often reached after a small number of groups.

A carefully crafted seeding statement was presented to individuals in each group, who were then asked to work alone for 5 minutes to generate ideas in response to the statement. The seeding statement for patients was: 'Thinking as broadly as you can, generate statements about *how low back pain affects your life (considering both yourself and those around you)*'. For the health professional group, the seeding statement was slightly different: 'Thinking as broadly as you can, generate statements about *how low back pain affects the life of people with the condition and the community*'. Participants were asked to write down their responses according to the following rules: one idea per statement, use bullet points, make the statements brief, and work alone. The nominal group technique uses a facilitator who then asks that the ideas be presented to the group in an egalitarian manner, whereby each participant in turn presents one item on their list, starting with the first, until all items have been presented. Participants were discouraged from passing judgments about the statements but were encouraged to seek clarification of the nature or content of the statement if necessary. The critical advantage of this approach is that the perspective of individuals is collected in a manner that is not influenced or biased by the researcher nor influenced by other, and at times dominant, group members.

Once all statements had been presented, participants were asked to sort the statements into conceptually similar groups according to any system that made sense to them. For this step, they were asked to work alone. MDS and cluster analysis were then used to process participants' input and generate two-dimensional maps of key concepts related to low back pain impact and the interrelationships among these clusters.

Participants were asked to independently consider and label each group of statements and to check that each of the statements fit within that group. If a statement or statements were not considered to fit within the group, participants were asked to nominate the appropriate grouping. They were also asked to consider whether any of the groups should be joined. After this had been completed on an individual basis, we again used a nominal group approach to organize the final groupings, their labels and the included statements. We also checked for any missing domains/concepts.

### Concept mapping with international experts

A similar concept mapping exercise was conducted via email and through a face-to-face workshop at the 10th International Forum for Primary Care Research on Low Back Pain held in Boston in 2009. The expertise of the expert international group was broad and included primary care, rheumatology, occupational health, physiotherapy, chiropractics, epidemiology, public health and health policy.

Prior to the Forum, an email was sent to all participants who had been allocated to the workshop (*n *= 31) asking them to generate statements in response to a similar seeding statement: 'Thinking as broadly as you can, generate statements about ... *how low back pain affects the life of people with the condition and those around them'*. Forty-five percent (14/31) of participants responded to this task.

The statements from the patient group, from the clinician/health policy group and from the Forum workshop participants were then combined and redundancies were removed. This final set of statements were then sent to Forum participants in a second email requesting that they sort the statements into conceptually similar groups according to any system that made sense to them. They were also asked to rank each of the statements in order of importance. Fifty-eight percent (18/31) completed this task.

The same process of multidimensional scaling and cluster analysis was used to process participants' input and generate two-dimensional maps of key clusters of low back pain impact and the interrelationships among these clusters.

At the Forum we presented the results of the patient and clinician/health policy-maker workshops and the final concept map that was generated by the Low Back Pain Forum workshop participants. Participants were asked to independently consider and label each group of statements and to check that each of the statements fit within that group. If a statement or statements were not considered to fit within the group, participants were asked to nominate the appropriate grouping. They were also asked to consider whether any of the groups should be joined. After this had been completed on an individual basis, the group worked together to organize the final groupings, their labels and the included statements. We also checked for any missing domains or concepts.

### Integration of the three concept maps

At this point we had three concept maps: two from the initial groups and one from the international expert group. The process of integrating the three maps included a number of steps. In addition to the two-dimensional MDS that underlies the concept maps, we undertook three-dimensional and four-dimensional MDS using the Clustan software [27] and repeated the cluster analysis on the outputs of these analyses. Sometimes a three-dimensional or four-dimensional MDS can more accurately capture the similarities between statements and leads to cleaner (more self-evidently homogeneous) clusters. The output of the MDS and cluster analysis is viewed as a tree diagram; a diagram that allows all cluster solutions from a single cluster to a number that equals the number of items to be examined. This diagram allows us to examine the division of items each time a cluster is split into two smaller clusters to determine whether this split has substantive meaning. Through this process we looked to determine the smallest number of clusters (most general concepts) that made sense, the largest number of clusters (most refined concepts) that made sense, and the items that are considered most typical of each refined concept.

At the level of the most general concepts, the results from different concept mapping groups tend to be similar. This means that the results can be combined at this level and the results from the different concept mapping groups provide different details under these high-level concepts. These results for each group analysis are displayed as mind maps (Mindjet Mind Manager software, 2010, MindJet Ltdr, Sydney, New South Wales, Australia). The mind maps are then combined so that the common general concepts form the first level of detail and the branches represent each substantively meaningful split identified through examination of the tree diagrams.

Throughout this process the researchers attempted to use the cluster names assigned by the original group participants. The mind map aims to provide a clear hierarchical overview of the burden of low back pain as seen by the participants. This hierarchical representation does not, however, show the richness of the relationships between the clusters as well as the original maps. For this reason, the integrated mind map needs to always be considered in conjunction with the original maps.

### Refinement of the structural model

The next step in refining the structural model was to check the proposed domains against the original item pool. The researchers classified every statement produced by the three concept mapping groups according to the proposed domains. In performing this classification we were looking for: items that cannot be classified - these may indicate the need for additional domains; items that seem to relate to more than one domain - these may be ambiguous items or may indicate a relationship between the hypothesized domains; domains that still seemed to contain multiple concepts and may need to be split; and match between domain names and the item content - a poor match may require renaming the hypothesized domain.

## Results

In response to the seeding statements, the three groups produced 305 statements: 47 from the patients, 61 from the stakeholders and 197 from the international expert panel (Table 1). Removing duplicates, the final set comprised 91 statements.

**Table 1:** Number of participants contributing to concept mapping and the number of statements produced

	Number of participants	Number of statements
Low back pain patient group	8	47
Stakeholders	10	61
Low Back Pain Forum workshop participants	14 generated statements	197
	18 completed sorting and rating of final set of statements^a^	91^a^

^a^The final set of 91 statements was derived by combining statements from all three groups omitting redundancies.

**Figure 1:**
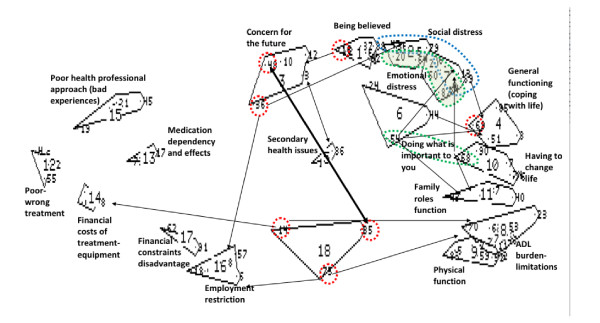
**Concept mapping results from Low Back Pain Forum Conference, Boston**. ADL, activities of daily life.

Figure 1 shows the concept map produced by the international expert panel. Each of the bounded shapes represents a cluster of statements. The large number is the cluster number designated by the software (that is, cluster 1, cluster 2, and so forth). The small numbers within each cluster represent the statements produced by the group in the nominal group phase (that is, each statement is given a number as it is entered into the program). In interpreting a concept map, it is usually best to work systematically around the edge of the map and then look at the central clusters. The items circled by dashed lines were considered to relate strongly to other items; this is inevitable when there are many ways of thinking about a concept.

Some of the most notable features of the map shown in Figure 1 are: the large number of statements related to the interaction between the reactions of others and the person's psychological state (seen in the top right-hand corner); the variety of statements related to the effort of living (down the right-hand side), which range from having to think about and plan daily activities and the physical weariness of many activities to having to make enduring changes in lifestyle; the burden related to peoples' interactions with societal institutions, including workplaces and treatment services (left-hand side); and the concepts that have both individual and health service aspects, such as effects of treatment and health states (central clusters). The maps produced by the other groups had a similar range of concepts and a similar emphasis on issues associated with the reactions of others and the effort of daily living.

**Figure 2:**
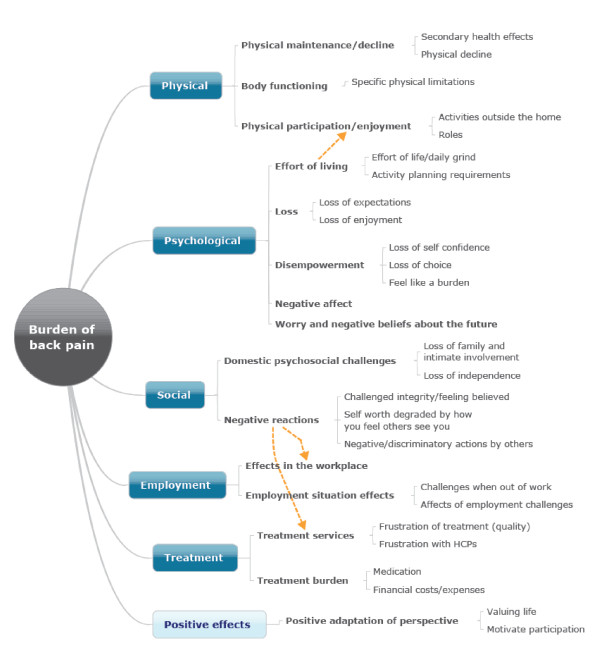
**Hierarchical model of low back pain burden (integrated from all concept maps)**. HCP, healthcare practitioner.

The next step involved the examination of the tree diagrams related to each of the concept maps to identify the minimum number of clusters that made sense, the maximum number that made sense, and representative statements - each of which was represented as a mind map. The result of this process is presented in Table 2, which in turn was used to hypothesize a set of major domains and subdomains and the structural model presented in Figure 2.

**Table 2:** Clusters, subclusters and representative statements

Clusters and subclusters	Representative statements
Psychosocial	
Loss	
Loss of expectations	Limitations on fulfillment of goals in life
Loss of enjoyment	Loss of enjoyment in life
Loss of self-confidence	Low self-esteem, especially from loss of roles
	Feel helpless when people stop you doing things
Negative affect	Irritation, anger and frustration
Worry and negative beliefs about the future	Worry about the future
	Fear that severe back pain will occur again
Global malaise	
Secondary health effects	Difficult to address other health issues
	May lead to weight gain
Effort of life/daily grind	Tiredness
	Makes you feel old
	Loss of motivation in life
Executive challenge	Always having to think about what you can and cannot do
Domestic psychosocial challenges	
Loss of family and intimate involvement	Left out of family activities
	Difficulty caring for others
Loss of independence	Need to ask for help to do things
Negative reactions^a^	
Challenged integrity/feeling believed	May be seen as a malingerer
	Wrongly considered lazy by others
Self-worth degraded by how you feel others see you	Always trying to hide pain from family so they do not worry
	Feel like a burden on workmates
Negative/discriminatory actions by others	Bullied by others
	May lose friends
Physical	
Functioning outside the home	Makes it hard to travel
	Leisure activities are limited
Specific physical limitations	Daily living is hard including basic self-care
	Difficulty lifting things
	Hard to sit
General physical impact	More and more physically unfit
	Fall easily
Treatment	
Treatment services	
Frustration of treatment (quality)	Waste time and money on dubious treatments and practitioners
	Unnecessary surgery and the problems this causes
Frustration with healthcare providers	Doctors not understanding there is anything wrong with you
	Back pain can make you distrust the medical profession
Condition burden	
Impact on others^b^	Need help from carers
Medication	Dependent on more and more medication
	Side-effects
Financial costs/expenses	Costs of treatment and equipment (necessary and unnecessary)
Employment	
Challenges when out of work	Difficult to get back to paid employment
	Reduced employment options, now and for the future
Challenges when working^c^	Many limitations on what tasks can be done
Effects of employment challenges	Difficult to get health insurance
	Reduced income resulting in poverty

^a^See also Employment. ^b^See also Challenges when working, Psychosocial. ^c^See also Impact on others.

As shown in Table 2 we identified four clusters (Psychosocial, Physical, Treatment and Employment), and each cluster included a variable number of subclusters. For example, within the Psychosocial cluster there were six subclusters including loss, negative affect, worry and negative beliefs about the future, global malaise, domestic psychosocial challenges, and negative reactions.

**Figure 3:**
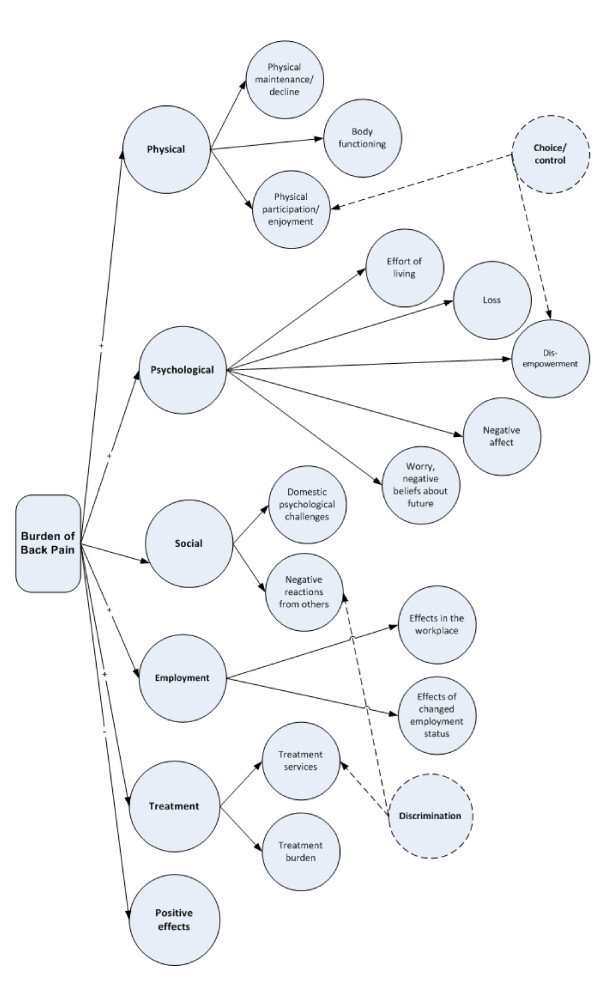
**Hypothesized, *a priori *measurement model to be tested with construction and validation samples**.

Figure 3 refines the hierarchical model developed from the mind map (Figure 2), to further hypothesize latent variables that are represented by a number of candidate items (derived from the concept mapping groups). The circles in Figure 3 each represent a hypothesized latent variable. In this model we hypothesize that there are six major domains, some of which have subdomains (up to five) and some of which do not. Given item content, we also hypothesize two further independent domains: Choice or control, which will be related to elements within the physical and psychological domains; and Discrimination, which will be related to elements within the social and treatment domains.

## Discussion

### Validity-driven instrument development

Our approach to construct definition and instrument development is based on the tenet that construct validity needs to be the primary concern of all instrument development activities and of all proposed applications of instruments. This is consistent with the descriptions provided by Pedhazur and colleagues [28], and the Standards for Educational and Psychological Testing developed jointly by the American Educational Research Association, the American Psychological Association and the (American) National Council on Measurement in Education [29,30]. The Standards describe validation as an ongoing process that commences with the conceptualization and continues each time someone proposes an additional interpretation or application of the tool [29].

While it is common practice in health research to refer to a tool as either validated or unvalidated, it is not tools but only their interpretations and applications that are validated. To maximize the likelihood of producing valid data in relation to a range of possible interpretations and applications of a tool, there are development processes that seek to protect the instrument against two categories of error; measuring less than the proposed construct (construct underrepresentation) or measuring more (construct irrelevant variance) [29]. Protection against the first type of error requires rigor in the processes of conceptualization and definition and the identification of a range of indicators. Protection against the second type of error requires rigor in psychometric analysis. We believe that three disciplines help achieve this necessary rigor: the use of grounded approaches for construct definition; the development of *a priori *structural hypotheses (that define relevant versus irrelevant variance); and the development of *a priori*, relational hypotheses as a basis for future construct validation.

The Standards contain 24 standards related to validity of a measure, but the first four of these specifically relate to the linkage between validity and possible interpretations (Table 3). It is clear that the authors of the Standards place a significant onus of responsibility on the developers of instruments to clarify the interpretations that are supported by available evidence at any point in time.

**Table 3:** Standards relating validity to interpretations

Standard 1.1	A rationale should be presented for each recommended interpretation and use of test scores, together with a comprehensive summary of the evidence and theory bearing on the intended use or interpretation
Standard 1.2	The test developer should set forth clearly how test scores are intended to be interpreted and used. The populations(s) for which a test is appropriate should be clearly delimited and the construct that the test is intended to assess should be clearly described
Standard 1.3	If validity for some common or likely interpretation has not been investigated, or if the interpretation is not consistent with the available evidence, that fact should be made clear and potential users should be cautioned against making unsupported interpretations
Standard 1.4	If a test is to be used in a way that has not been validated, it is incumbent on the user to justify the new use, collecting new evidence if necessary

An important initial step in scale development, and the final step in development of the hypothesized model, involves writing (hypothesized) descriptors about characteristics of people with a high score and people with a low score on scales related to each hypothesized domain. This exercise helps to clarify whether the domain can be represented as a scale or whether it is simply a checklist of possible characteristics, the desired range of item difficulty, and possible relationships between scale scores and other variables (other scales, demographic and clinical variables, outcomes of interventions). This final point is an important and often neglected step in preparing for construct validation by developing a broad range of *a priori *hypotheses about the behavior of the scales in relation to other variables (the so-called nomothetic web) [28,31].

In considering the ongoing process of validation once the instrument has been developed, it is necessary to specify the interpretations and applications that we are seeking to validly and reliably achieve. These interpretations and applications are presented in Table 4, together with some of the evidence - or the processes to obtain the evidence - that is required to support validity of each type of interpretation/application. Table 4 shows that the expansion of valid interpretations and applications occurs in a number of stages that build upon each other. The first two stages are integral to the process of psychometric development through the application of draft tools to a construction and validation sample (see below). Evidence in relation to the second two proposed applications accrues through use of the tool, while the step from interpretation of data at the group level to interpretation at the individual level usually requires additional technical analysis as well as a body of evidence about the meaning and behavior of each scale acquired through widespread use with groups. There are also steps that can be taken during the psychometric development phase to increase the likelihood that the tool will be usable with individuals. These steps relate to ensuring that the scales have certain properties in relation to the range of difficulty that the items cover and the extent to which they can give scores spread evenly across this range. While the meaning of difficulty is clear in academic tests in this situation, a difficult item would be one where few people give the most positive possible response. There are also different reliability requirements related to each level of use, with individual applications having the most stringent requirements.

**Table 4:** Proposed interpretations/applications and evidence required to support the measure's validity for low back pain burden

Proposed interpretation/application	Evidence of validity or activities to obtain this evidence
**Interpretations/applications applied to groups - supported through initial development processes**	
Describe the burden of low back pain on a set of scales that reflects the full range of the experience of people with low back pain	Thorough, grounded identification of the range of issues that contribute to low back pain burden
	Iterative process of organizing these into domains and potential scales
	Comparison with interview data at a number of stages of development
Quantify variations in the effects of low back pain across a broad range of sufferers on a range of scales	Cluster analysis to identify score profiles and qualitative confirmation of these
	Tests of structural invariance across groups
**Interpretations/applications applied to groups - supported through subsequent applications of the tool^a^**	
Describe the relative importance of different domains of low back pain burden in comparing one population with another (for example, needs identification)	Accumulated evidence about what is a high average score and what is a low average score for each scale^b^
	Establishment of whole of population norms and subgroup norms
	Tests of structural invariance
Validly assess changes in low back pain burden in a group over time or as a result of interventions	Application for a range of evaluation purposes including comparison with other subjective and objective indicators of change
	Development of estimates of meaningful change
**Interpretations/applications applied to individuals**	
Assess the relative needs of an individual with low back pain across a range of domains	Attention to item scaling properties during psychometric development
	Comparison with other subjective and objective indicators of status
Measure changes in individuals over time or in response to interventions	Comparison with other subjective and objective indicators of change
	Development of estimates of meaningful change

^a^Two further possible applications are the development of an overall score to compare the total burden of one population group with another, and also the attachment of utilities to scores to enable comparisons with other conditions. At this stage we do not propose to pursue either of these applications. ^b^It is important to note that we do not assume the scales all scale equally or that an average score of 3.5 on one scale will necessarily indicate a greater problem than a score of 4 on another scale. These relative weightings are necessarily dependent on the values of individuals or on some estimate of the average values of groups and populations.

### Implications for the measurement of the burden of low back pain

One of the primary reasons for conducting this research was the observation that existing instruments inadequately capture the range of impacts of low back pain that are commonly reported by people with low back pain and the clinicians that work with them. This project has produced a conceptual framework that includes many concepts not included in the tools most commonly used to assess needs and/or outcomes for people with low back pain.

At one end of the spectrum, because low back pain has until recently been thought mainly a work-related problem, outcome measures have often been limited to occupational aspects of burden: most of all, measures of absence from work, and the consequent financial costs. Such measures only capture part of the burden of low back pain.

At the other end of the spectrum, Deyo and colleagues proposed a core set of six indicators for routine clinical use that included pain symptoms, function, well-being, disability, social role and satisfaction with care [32]. Another core set of measures proposed for evaluating the effectiveness of treatment in clinical trials and routine care was proposed by Bombardier [33]. Recognizing the importance of the patient's perspective, she proposed the following five domains: back-specific function, generic health status, pain, work disability, and patient satisfaction [33]. Similar to these proposals, the Initiative on Methods, Measurement, and Pain Assessment in Clinical Trials group recommended a core set of six outcome domains be considered in chronic pain clinical trials: pain, physical functioning, emotional functioning, participant ratings of global improvement and satisfaction with treatment, symptoms and adverse events, and participant disposition [34].

More recently, Kopec and colleagues proposed a web-based computerized adaptive test (CAT-5D-QOL) to measure five domains of health-related quality of life (Daily Activities, Walking, Handling Objects, Pain or Discomfort, and Feelings) for patients with back pain based upon item banks developed for these domains relevant to arthritis [35]. Many measures have been developed to specifically quantify the limitations that low back pain places upon functional status. For example, in a 2004 systematic review Grotle and colleagues identified a total of 36 back-specific questionnaires [36]. The authors classified the content of the questionnaires based upon the World Health Organization's International Classification of Functioning, Disability and Health (ICF); they found that while most of the questionnaires had a focus on activity limitations, there was a wide variation in their underlying constructs and content. Many questionnaires also included constructs of pain and symptoms, sleep disturbances, psychological dysfunction, physical impairment and social functions.

The brief and comprehensive ICF core sets for low back pain, based upon the ICF framework, are further attempts to develop a standardized set of indicators to encompass the key functional problems of patients with low back pain envisaged to be used for a variety of purposes including clinical studies and multidisciplinary assessment in clinical care [37]. These were formed by consensus among a group of international clinical experts comprising physicians, occupational and physical therapists, who integrated evidence from a Delphi exercise to identify the most relevant ICF categories in patients with chronic health conditions including back pain [38], a systematic review to identify the concepts contained in outcome measures in clinical trials of musculoskeletal disorders and chronic widespread pain [39], and a study in a convenience sample of people undergoing rehabilitation for one of several chronic conditions including low back pain who were administered the ICF checklist [40]. The comprehensive and brief ICF core sets include 78 and 35 categories, respectively, which cover not only aspects related to pain but also a wide spectrum of activities, social and environmental factors that affect functioning. In keeping with our conceptual model, these core sets recognize the importance of support and relationships, attitudes of significant others and health professionals as predictors of disability in people with low back pain.

A Norwegian study in a convenience sample of 118 patients with low back pain, however, has identified gaps in the comprehensive ICF core set with respect to capturing problems of importance to patients [41]. This study compared the relationship between health problems rated by health professionals using the comprehensive ICF core set and patients' self-reported health problems identified by the Oswestry Disability Index and the World Health Organisation Disability Assessment Schedule II. Relevant domains not covered by the ICF included the subjective domain related to the impact of back pain and the feeling of being a burden to their family, while problems with sexual functions and relationship were poorly reflected in the health professionals' assessments.

Our model for the measurement of the burden of low back pain aims to comprehensively capture all of the various impacts of this condition on the individual. The model includes several domains that have not until now been considered important to measure in patients with low back pain, although they may contribute significantly to the individual's burden; for instance, loss of independence, worry about the future, negative or discriminatory actions by others, and secondary health effects, among others.

The new tool will have a wide range of potential applications for researchers, clinicians, policy-makers and insurance agencies; and for a range of purposes, including needs identification, service planning, evaluation, research and, eventually, for individual clinical assessment and monitoring. In suggesting such a range of applications, we are aware of our responsibility to consider the evidence for validity in relation to each interpretation and application [29,30].

To strengthen potential generalizability, we have used both a local approach and an international approach to scope and define low back pain burden, nominal group approaches and concept mapping. The questionnaire is being developed with input from an international team of experts in the field. To facilitate comparison of the burden of back pain between countries and between studies, steps are being taken to ensure its wide applicability and cross-cultural generalizability.

In assessing health priorities, allocating resources, and evaluating the potential costs and benefits of public health interventions, governments often consider the burden of a disease and its contribution to the overall health of the population. Information obtained from a single comprehensive measure of back pain burden will greatly enhance research efforts to identify major determinants of back pain burden and population groups that are most affected and to ensure efficient allocation of resources. This information may also inform the development and evaluation of novel new interventions that could improve patient-relevant outcomes.

While the measurement model (Figure 3) does test for a single underlying latent variable, which we have called the burden of low back pain, we expect the questionnaire will be used as a multidimensional tool providing a profile of scores across the various scales. We will not be attempting to provide a scoring mechanism to gain a single overall score. In our experience it is more useful to be able to use profiles of scores to describe the needs of different patient groups and to distinguish the benefits of different types of interventions than to generate a global indicator that is at such a high level of abstraction no-one will be clear what it means. A profile of scores will also serve to highlight the critical psychosocial aspects of the burden of low back pain that have not been adequately addressed in existing tools. It is hoped that this profile of scores will support a greater clinical emphasis and increased research focus on these aspects of the burden experienced by people with back pain.

## Conclusions

The present paper has described the process of developing a strong, *a priori *hypothesis of a measurement model for a multidimensional measurement of the burden of low back pain. The model will now be tested with a sample of approximately 600 people and may be refined on the basis of structural equation modeling analysis of the data. The refined tool will be retested on a separate (validation) sample of another 600 people. These are all foundational steps in a process of establishing construct validity for an expanding range of applications of the tool.

This paper has demonstrated how the application of a rigorous set of disciplines -by which grounded consultation and conceptualization processes lead to strong *a priori *hypothesis relating to measurement - provides a firm foundation for building the evidence of validity for a wide range of potential interpretations and applications. The conceptualization process has led to a much richer and more extensive set of concepts relevant to assessing the needs of people with back pain than is captured in the outcome tools previously applied.

## Abbreviations

ICF: International Classification of Functioning Disability and Health; MDS: multidimensional scaling.

## Competing interests

The authors declare that they have no competing interests.

## Authors' contributions

RBu and RHO conceived the study, contributed to its design and coordination, and drafted the manuscript. RBa contributed to the design of the study, performed the statistical analysis, and drafted the manuscript. GE provided input on the statistical analysis. CED and EI assisted with the international expert workshop. All authors contributed to the interpretation of the findings and read and approved the final manuscript for publication.
